# Nef-induced CCL2 Expression Contributes to HIV/SIV Brain Invasion and Neuronal Dysfunction

**DOI:** 10.3389/fimmu.2019.02447

**Published:** 2019-10-15

**Authors:** Michael H. Lehmann, Jonas M. Lehmann, Volker Erfle

**Affiliations:** ^1^Institute of Virology, Technische Universität München, Munich, Germany; ^2^Institute for Infectious Diseases and Zoonoses, Ludwig-Maximilians-Universität München, Munich, Germany; ^3^Department of Informatics, Technische Universität München, Munich, Germany

**Keywords:** AIDS, astrocyte, autophagy, chemokine, dementia, inflammation, neuron, virus

## Abstract

C-C motif chemokine ligand 2 (CCL2) is a chemoattractant for leukocytes including monocytes, T cells, and natural killer cells and it plays an important role in maintaining the integrity and function of the brain. However, there is accumulating evidence that many neurological diseases are attributable to a dysregulation of CCL2 expression. Acquired immune deficiency syndrome (AIDS) encephalopathy is a severe and frequent complication in individuals infected with the human immunodeficiency virus (HIV) or the simian immunodeficiency virus (SIV). The HIV and SIV Nef protein, a progression factor in AIDS pathology, can be transferred by microvesicles including exosomes and tunneling nanotubes (TNT) within the host even to uninfected cells, and Nef can induce CCL2 expression. This review focuses on findings which collectively add new insights on how Nef-induced CCL2 expression contributes to neurotropism and neurovirulence of HIV and SIV and elucidates why adjuvant targeting of CCL2 could be a therapeutic option for HIV-infected persons.

## Introduction

Acquired immune deficiency syndrome (AIDS), caused by the human immunodeficiency virus (HIV) (1, 2), has to date resulted in the deaths of over 32 million people. According to the 2019 UNAIDS Global AIDS Update, 1.7 million people became newly infected with HIV in 2018 resulting in a total number of 37.9 million people living with HIV worldwide. To date, there is no effective protective vaccine against HIV or even a feasible cure available for HIV-infected patients (3, 4).

In the mid-1990s combined anti-retroviral therapy (ART) was introduced, which considerably reduced the mortality of HIV-infected patients. However, since then, the prevalence of HIV-associated diseases has increased. A major obstacle toward the development of therapies against these diseases that affect a number of organs such as the heart, lungs, kidneys, and the brain is due mainly to the fact that the disease pathogenesis is poorly understood (5–8). In the meantime, there exists at best a consensus that a systemic and persistent activation of the immune system plays a major role in the disease pathogenesis (9–11). Moreover, it is difficult to accurately differentiate between age-related neurodegeneration, other neurodegenerative diseases and HIV-associated neurocognitive disorders (HAND) (12). However, attempts have been made to identify biomarkers to diagnose neurocognitive impairment in HIV-infected persons and activated monocytes/macrophages and C-C motif chemokine ligand 2 (CCL2) appear to be the most promising amongst them (13).

CCL2, also named monocyte chemoattractant protein 1, is a chemotactic cytokine for monocytes (14) and T cells (15), which are the main target cells of HIV-1. CCL2 decreases interferon-alpha expression (16), and promotes HIV/SIV replication by up-regulation of surface C-X-C motif chemokine receptor 4 expression (17).

CCL2 binds to the C-C motif chemokine receptor 2 (CCR2), which is expressed by neurons (18), human fetal astrocytes (19) and brain microvascular endothelial cells (BMECs) (20). CCL2 also binds to the D6 chemokine decoy receptor, which is expressed on adult human astrocytes (21).

The CCL2-CCR2 axis has been shown to play a key role in multiple sclerosis and in experimental autoimmune encephalomyelitis (22), in addition to exacerbating neuronal damage after status epilepticus (23), eliciting itch- and pain-like behavior in allergic contact dermatitis (24), as well as mediating alcohol-induced neuroinflammation and neurotoxicity (25).

## HIV and SIV Associated Dementia, Encephalitis and Neuronal Damage

Without combined ART, HIV causes dementia which is characterized by deficiencies in cognition, motor disorders, and behavior abnormalities (26). Pathological manifestations of HIV-associated dementia (HAD) appear as meningitis, encephalitis and vacuolar myelopathy (27). A similar clinical picture has been observed in the SIV/macaque model (28, 29). Even after the introduction of combined ART, HAND remain (8), and, in fact SIV-infected macaques treated with suppressive ART also show ongoing neurodegeneration and inflammation (30). The reason for this phenomenon is unknown although several explanations have been proposed, e.g., that anti-retroviral drugs cannot access the central nervous system (CNS), are not effective in eliminating viral reservoirs, or themselves contribute to HAND (31, 32). However, since specific CNS-targeted ART failed to improve neurocognition in HIV-infected patients compared to non-CNS-targeted (33), it has been hypothesized that early events after primary infection with HIV/SIV are critical for initiating the development of HAND (8).

Indeed, SIV was detected in the brains of macaques within a few days after intravenous infection (34, 35). Further, HIV nucleic acid was detected in the brain of an HIV naïve patient who died 15 days after intravenous inoculation of indium-111-labeled white blood cells, which originated from an HIV-infected individual (36). Additionally, a more recent study showed that HIV RNA is present in the cerebrospinal fluid (CSF) of humans as early as 8 days after HIV infection (37). This suggests that HIV/SIV is capable of exploiting a distinct mechanism to enter the brain rapidly.

Entry of SIV into the brain and induction of neuropathology does not appear to depend on a sustained high viral load because the SIVmac32H(pC8) strain, whose replication is attenuated *in vivo* (38, 39), was detected in the brain 3 days after infection of macaques where it caused persisting neuroinflammation (40). The attenuated phenotype of SIVmac32H(pC8) is most probably due to a 12 base-pair deletion in its *nef* gene, which results in an in-frame deletion of the amino acids 143–146 of the translational product (38). Although this Nef variant was detected at lower levels *in vitro* compared to other variants (41), it was definitely detected in the brain of macaques infected with SIVmac32H(pC8) (42). However, SIV strains containing nucleotide deletions in the *nef* long-terminal repeat (*nef* /LTR) overlap region, analogous to the HIV strain of the Sydney blood bank cohort (SBBC), could not be detected in the brains of macaques despite viral replication in the periphery (43). Of note, members of the SBBC who had become infected with an HIV strain containing the nucleotide sequence deletions in the *nef* /LTR region that results in a truncated Nef protein of 24 amino acids (44), did not or only slowly progressed to AIDS including HAD (45).

## The Nef Protein of HIV/SIV: Importance for AIDS Progression and Its Intercellular Transfer

The importance of Nef for AIDS progression was confirmed in SIV-infected rhesus monkeys and HIV-transgenic mice (46, 47). Additionally, it was shown that Nef is required for high viral load *in vivo* (47). These findings have stimulated a series of studies aiming to identify the mechanistic background with the ultimate goal to exploit the knowledge for therapeutic intervention. Indeed, numerous cellular interaction partners and pathophysiological functions of Nef have been detected (48, 49), and several models of how Nef executes its role in HIV/SIV replication and immunopathogenesis have been proposed (50). In 2009, Kyei et al. showed that HIV Nef inhibits autophagic maturation in human macrophages and thereby provided a convincing explanation of how Nef acts at the molecular level to enable efficient replication of HIV (51). Inhibition of autophagy increases the production of proinflammatory cytokines (52, 53) including CCL2 (54, 55). Thus, Nef also seems to contribute to chronic inflammation, which occurs in HIV-infected persons (56).

HIV Nef was found in supernatants of *nef*-expressing BHK cells (57), yeast (58), and HEK293 cells (59), which was surprising at the time of these discoveries because *nef* does not code for an N-terminal signal sequence that would direct the protein to the cell secretory pathway leading to export. Thus, the mechanism by which Nef is released from infected cells was regarded as an open question. In the past, on analyzing the supernatants of BHK cells infected with recombinant vaccinia virus expressing HIV Nef, it was assumed that Nef could be released by vesicles (57). Today, it is recognized that not only proteins but also lipids and RNA can be released from a cell by extracellular vesicles (60).

In 2003, it was shown that HIV Nef induces an accumulation of multivesicular bodies (MVBs) and that Nef itself is present in MVBs (61). MVBs can fuse with the cell plasma membrane, leading to the release of 40–90 nm diameter vesicles, termed exosomes, into the extracellular environment (62). Consequently, it was tempting to speculate that Nef could be released from cells by exosomes. However, it was challenging to test this hypothesis in HIV-infected cells because Nef is incorporated in virions (63, 64).

The astrocytoma cell line TH4-7-5 is persistently infected with the HIV isolate TH4-7-5 which has a mutation in the *nef* gene (GenBank accession number: L31963.1), resulting in a myristoylation-deficient Nef (65). However, myristoylation of Nef is required for optimal HIV replication *in vitro* (66). Thus, a myristoylation-deficient Nef and a block in HIV Rev function most probably effected a very low production of infectious virus but a high production of Nef in astrocytoma TH4-7-5 cells (65, 67). We took advantage of the astrocytoma cell line TH4-7-5 and examined whether Nef is present in the supernatants of these cells. Application of a two-step centrifugation protocol, previously shown to enable the enrichment of microvesicles including exosomes from cellular supernatants (68), resulted in the detection of Nef in the pellets of centrifuged supernatants of these cells (69).

It was later confirmed that Nef is released from HIV-infected cells (70, 71) by microvesicles and it was even claimed that this occurs via exosomes (72). However, there is still an ongoing debate regarding the type of vesicle by which Nef leaves the cell (73, 74). Further, Nef was detected in microvesicles and exosomes isolated from the plasma of HIV-infected persons despite them receiving ART, and it has been shown that exosomes derived from HAD patients can transfer *nef* mRNA to cells, leading to Nef expression and subsequent induction of cellular genes (75, 76).

Nef was also found in uninfected human peripheral blood mononuclear cells (PBMCs), which can transfer Nef to human umbilical cord vein endothelial cells (77). A recent study not only reported that Nef is released by vesicles from HIV-infected cells, but also confirmed the result for SIV-infected cells and has additionally shown that extracellular vesicles containing Nef circulate in the blood of SIV-infected macaques (78). Meanwhile, the process of protein and mRNA transfer by exosomes and other extracellular vesicles even between different types of cells is well understood (79). In summary, irrespective of the type of extracellular vesicle from which Nef is released by HIV/SIV infected cells, Nef is present in the extracellular environment independently of virions and can enter uninfected cells where it affects cellular functions and gene expression.

Additionally, cells can exchange molecules and organelles directly via tunneling nanotubes (TNTs), which are about 50–200 nm long thin actin rich membrane conduits, even between different types of cells (80). Nef can induce TNT formation (81, 82), and it can also be transferred to B cells via TNTs from HIV-infected macrophages (83), from macrophages to T cells (82), from *nef*-expressing T cells to hepatocytic cells (84) and also between macrophages (81). Importantly, Nef is transferred from T cells and monocytes to human coronary arterial endothelial cells via TNTs, leading to apoptosis and CCL2 expression (85).

## Nef-induced CCL2 Expression and the Function of the Blood-Brain-Barrier

CCL2 increases the blood-brain barrier (BBB) permeability (86, 87), and much progress has been made in revealing the molecular mechanism of how leukocytes, governed by CCL2, pass the BBB (88). Therein, astrocyte and BMEC-derived CCL2 play complementary roles (89).

A natural repair mechanism to restore damaged brain tissue after experimentally-induced ischemia starts with the recruitment of CCR2^+^Iba1^+^ monocytes from the periphery, which then differentiate into brain Iba1^+^NG2^+^ cells within the brain parenchyma (90, 91). Transmigration of CCR2^+^Iba1^+^ monocytes through the BBB is enabled by a transient expression of CCL2 in astrocytes and endothelial cells that lasts for only 2 days (92). Indeed, under normal physiological conditions, the BBB is impermeable for circulating monocytes (93, 94), and therefore invasion of a healthy brain by HIV/SIV should not happen as fast as it has been observed. But a specific HIV/SIV-triggered mechanism leading to CCL2 expression in BMECs may enable HIV/SIV to get access to the brain either in the form of free virions or via infected CCR2^+^ cells. In this respect, it was significant to observe that Nef (i) can be transferred from human PBMC to human endothelial cells (77), (ii) was detected in endothelial cells of *nef*-transgenic mice and macaques infected with SHIV-nefSF33, and (iii) induces CCL2 expression in endothelial cells (85).

Invasion of the brain by leukocytes would additionally require an upregulation of adhesion molecules on both endothelial and infected cells. Indeed, it has been shown that HIV Nef upregulates the intercellular adhesion molecule 1 (ICAM-1) in vascular endothelial cells (95). ICAM-1 interacts with the lymphocyte function-associated antigen 1 (LFA-1), and its subunits, CD11a and CD18, are upregulated in HIV-infected monocytes (96, 97). Endothelial-derived CCL2 activates CD11a, leading to a firm arrest of monocytes on endothelial cells (98, 99), and mediates the subsequent transendothelial migration (100).

**Figure 1:**
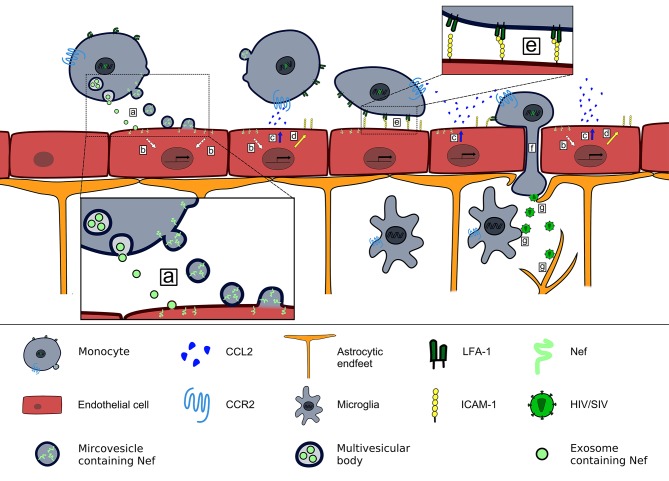
Contribution of Nef-induced CCL2 expression to HIV/SIV neurotropism. HIV/SIV infected monocytes release microvesicles and exosomes that transfer Nef into brain endothelial cells (a), where Nef induces a signaling pathway (b) that leads to release of CCL2 at the luminal side of the BBB (c) and upregulation of ICAM-1 (d). CCL2 binding to CCR2^+^cells triggers a conformational change of LFA-1 that enables their firm adhesion to brain endothelial cells via LFA-1–ICAM-1 interaction (e). Endothelial CCL2 expression enables transendothelial migration of HIV/SIV-infected CCR2^+^monocytes into the brain parenchyma (101) (f). There HIV/SIV infects astrocytes and microglia (g).

In summary, the findings collectively result in a model in which Nef-containing PBMCs and extracellular vesicles carrying Nef attach to and transfer Nef into endothelial cells, leading to CCL2 production that can cause BBB leakiness and subsequent entry of HIV/SIV by infected cells into the brain (Figure 1). Of note, this provides a simple explanation of why SIV with a deleted *nef* gene cannot enter the brain (43).

## Nef-induced CCL2 Expression and Neuronal Dysfunctions

Once in the brain, HIV/SIV cannot be eliminated by ART. The virus persists and triggers a chronic inflammation leading to sustained leukocyte infiltration, astrogliosis and neuronal degeneration (102, 103). In brain tissues of HIV-infected patients, HIV DNA was detected in the cells of the macrophage lineage and in astrocytes, the most abundant cell type in the brain. However, it was not found in neurons (104), which is in accordance with the finding that perivascular macrophages and microglia, but not neurons, can be productively infected with HIV/SIV (105, 106). These findings indicate that an indirect mechanism causes neuronal dysfunction and damage, and microglia that release exosomes and microvesicles containing Nef (107) may play an important role therein. It has long been known that HIV and SIV antigens are present in astrocytes of primary infected tissues (106, 108). Recently, a hypothesis was proposed that explains this finding (109) and challenges the consensus that HIV/SIV can infect astrocytes (110).

Significantly, Nef is highly expressed in astrocytes (111), promotes replication of HIV (112), and is also released by exosomes (113) or any other extracellular vesicle (114). Human astrocytes infected with recombinant Sindbis virus vector encoding *HIV nef* produced elevated CCL2 mRNA levels, which was independent of the *nef* variant tested (115). Induction of CCL2 expression by HIV Nef was confirmed in U-251 MG astroglioma cells transfected stably with *nef* (116), in primary rat astrocytes *in vivo* (117), and in primary murine macrophages and microglia (118). Animal models have provided evidence that there is a direct link between Nef-induced CCL2 expression and neuronal dysfunction and damage. Macrophages expressing HIV Nef, which were implanted into the rat hippocampus, triggered immigration of monocytes/macrophages, tumor necrosis factor expression, and astrogliosis, a hallmark of HIV encephalitis (HIVE). In addition, the neurotoxicity triggered by Nef was associated with cognitive deficits (119). Cognitive deficits in particular spatial and recognition memory were observed in rat brains in which primary astrocytes were implanted that expressed HIV Nef. This was associated with Nef-induced CCL2 expression, which resulted in immigration of macrophages in the hippocampus and loss of hippocampal CA3 neurons in these animals (117). In transgenic mice, in which HIV Nef was expressed specifically in macrophages and microglia, CCL2 was increased in the brain, and the dopamine system was affected, leading to mania-like behavior, especially in males (118).

There are several studies demonstrating that increased CCL2 concentrations correlate with HAD/HAND. Elevated levels of CCL2 were detected in the CSF of HIV-infected individuals positively diagnosed with HAD (120, 121). Microglia and astrocytes of HIV-infected persons suffering from HIVE produce CCL2 (122), which was confirmed for SIV infected macaques (123). Additionally, a specific small nucleotide polymorphism in the CCL2 promoter, which leads to increased CCL2 expression and infiltration of mononuclear phagocytes into tissues correlates positively with the risk of HAD (124). Cocaine, known to exacerbate neurodegeneration in persons infected with HIV, induces CCL2 expression in microglia and leads to increased transmigration of monocytes into the brain (125).

It is now also known that CCL2 affects neurons directly in addition to enhancing the transmigration of infected leukocytes through the BBB (126). For example, over-induction of CCL2 in astrocytes causes dopaminergic neurodegeneration in 1-methyl-4-phenyl-1,2,3,6-tetrahydropyridine mice (127), and an inhibition of CCL2 expression protects neurons against amyloid-beta-induced toxicity (128). Indeed, CCL2 mediates cell death in neurons of the hippocampal CA3 region after kainic acid-induced seizures in mice. Neuronal degeneration was associated with behavioral impairment, memory decline, and anxiety (129), all characteristics which have been observed early after infection of humans with HIV and even in HIV-infected persons receiving ART (130–132).

**Figure 2:**
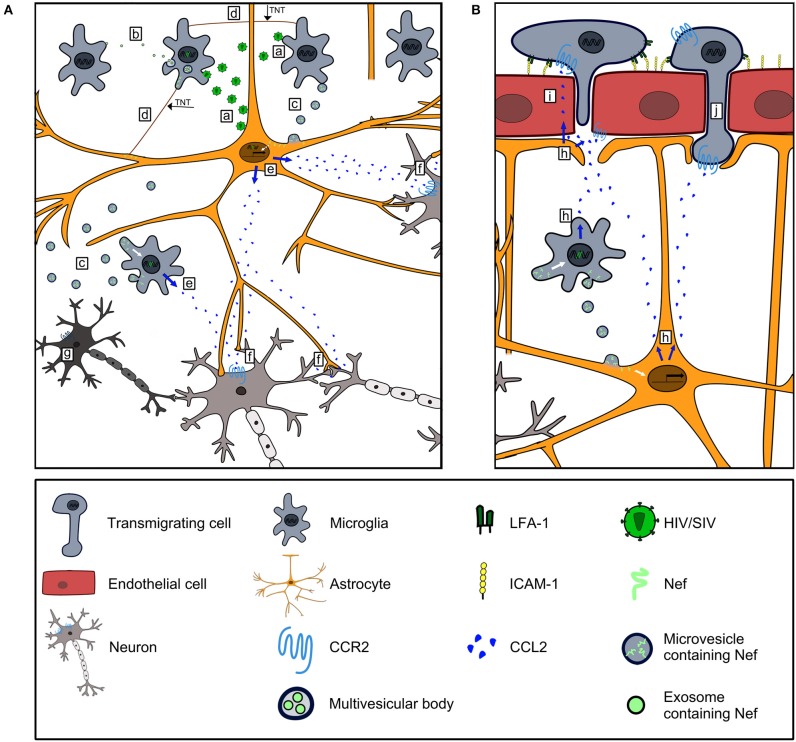
Contribution of Nef-induced CCL2 expression to HIV/SIV neurovirulence. **(A)** HIV/SIV-infected microglia and astrocytes infect uninfected microglia and astrocytes (a), and disseminate Nef via exosomes (b), microvesicles (c) and TNTs (d) to uninfected cells. Nef harboring astrocytes and microglia express CCL2 (e). CCL2 stimulates CCR2 signaling in neurons leading to their dysfunction (f) and death (g). **(B)** CCL2 produced by Nef harboring astrocytes and microglia (h) is transported transcellularly across BMEC (136) to act on CCR2^+^cells along the luminal side of the BBB (i). CCL2 binds to CCR2 on BMEC and mediates disruption of endothelial junctions (86, 87) to foster invasion of CCR2^+^cells into the brain (j).

CCR2, the receptor of CCL2, is present on neurons (18), and its absence reduced brain damage as well as BBB permeability in an experimental stroke model in mice (133). Similar to the process in an HIV/SIV infection, CCR2 plays a key role in the accumulation of myeloid cells in the brain and the activation of hippocampal myeloid cells upon infection with Theiler's murine encephalitis virus (TMV). Notably, CCR2 deficient mice had almost no hippocampal damage during TMV infection (134). Thus, CCL2 represents a convincing candidate to explain neuronal dysfunction and damage (135) which occur in HIV/SIV infected humans and animals (Figure 2). Additionally, CCL2 is major mediator of pain (137), and chronic pain is a common burden in people living with HIV/AIDS (138).

## Summary

The findings summarized herein not only integrate well into the “Trojan horse” model that states that a cell infected with HIV/SIV enters the brain leading to a persistent infection and consequently HAND (139) but also add to this model the fact that the transfer of Nef by microvesicles into endothelial cells and the subsequent induction of CCL2, mimics a pathophysiological state of the brain to which monocytes are recruited normally. Nef, in combination with other HIV/SIV proteins and even anti-retroviral drugs, possibly work together more efficiently to enable a rapid entry of HIV/SIV-infected cells into the brain (140). This interplay presumably plays a general role in HIV-associated diseases (141).

In the brain, HIV/SIV-infected cells such as astrocytes and microglia distribute Nef to uninfected cells via microvesicles and TNTs. Thereby, there is a steady increase in the number of Nef-bearing, non-infected cells which produce CCL2. HIV Tat in astrocytes seems to contribute to an increase in the levels of CCL2 in the brain (142, 143). The persistent non-physiological expression of CCL2 leads to sustained cell infiltration into the brain and a disturbance of neuronal functions. If a person is infected with HIV subtype B then Tat could enhance CCR2 activation through its acidic region (144, 145). Moreover, when present in sufficiently high concentrations in the brain, Tat could definitely exacerbate neuronal dysfunctions through its basic region (146). Moreover, besides CCL2, the C-X-C motif chemokine 10 (CXCL10) has also been identified as a biomarker for HAND (13), especially in HIV-infected women (147) and this chemokine can also be induced by Nef (115).

## Conclusion

The findings summarized here classify HIV/SIV Nef-induced CCL2 expression in the complex pathogenesis of HAND, and once again highlight the special role which the CCL2-CCR2 axis can play in a neurological disease. Consequently, drugs which have been developed to target this chemokine or its receptor could also be an option for an adjuvant therapy in HIV-infected persons.

## Author Contributions

ML wrote the initial draft. ML and VE discussed the manuscript. JL, ML, and VE edited the manuscript. JL and ML designed and drew the illustrations.

### Conflict of Interest

The authors declare that the research was conducted in the absence of any commercial or financial relationships that could be construed as a potential conflict of interest.
